# IDAC: Federated Learning-Based Intrusion Detection Using Autonomously Extracted Anomalies in IoT

**DOI:** 10.3390/s24103218

**Published:** 2024-05-18

**Authors:** Takahiro Ohtani, Ryo Yamamoto, Satoshi Ohzahata

**Affiliations:** Graduate School of Informatics and Engineering, The University of Electro-Communications, Chofu 182-8585, Japan; t.ohtani@net.lab.uec.ac.jp (T.O.); ohzahata@uec.ac.jp (S.O.)

**Keywords:** IoT, intrusion detection system, machine learning, anomaly detection

## Abstract

The recent rapid growth in Internet of Things (IoT) technologies is enriching our daily lives but significant information security risks in IoT fields have become apparent. In fact, there have been large-scale botnet attacks that exploit undiscovered vulnerabilities, known as zero-day attacks. Several intrusion detection methods based on network traffic monitoring have been proposed to address this issue. These methods employ federated learning to share learned attack information among multiple IoT networks, aiming to improve collective detection capabilities against attacks including zero-day attacks. Although their ability to detect zero-day attacks with high precision has been confirmed, challenges such as autonomous labeling of attacks from traffic information and attack information sharing between different device types still remain. To resolve the issues, this paper proposes IDAC, a novel intrusion detection method with autonomous attack candidate labeling and federated learning-based attack candidate sharing. The labeling of attack candidates in IDAC is executed using information autonomously extracted from traffic information, and the labeling can also be applied to zero-day attacks. The federated learning-based attack candidate sharing enables candidate aggregation from multiple networks, and it executes attack determination based on the aggregated similar candidates. Performance evaluations demonstrated that IDS with IDAC within networks based on attack candidates is feasible and achieved comparable detection performance against multiple attacks including zero-day attacks compared to the existing methods while suppressing false positives in the extraction of attack candidates. In addition, the sharing of autonomously extracted attack candidates from multiple networks improves both detection performance and the required time for attack detection.

## 1. Introduction

In recent years, the Internet of Things (IoT) has expanded rapidly in various fields such as healthcare, industry, and smart appliances, and has also become indispensable in our daily lives. However, information security challenges for devices deployed in IoT environments have become apparent due to their limited available resources derived from operational power and deployment cost constraints, that is, they are not equipped with sufficient resources to apply advanced security measures within the devices [1]. In fact, in 2016, there was an attack by the Mirai Botnet that targeted vulnerable IoT devices [2]. The Mirai Botnet launches attacks against specific IoT devices and initiates malware that communicates with a Command and Control (C2) server to form a botnet that is used to conduct large-scale Distributed Denial-of-Service (DDoS) attacks against its targets. Subsequently, Satori, a variant of the Mirai Botnet, emerged and formed a botnet by conducting zero-day attacks that mainly target unpatched vulnerabilities [3].

Zero-day attacks are attacks that exploit undiscovered vulnerabilities in software before vendors or others take measures. One of the countermeasures against zero-day attacks on IoT devices is the installation of a Network Intrusion Detection System (NIDS) on the IoT devices’ network to monitor network traffic and notify network administrators when signs of an intrusion are detected. NIDS is generally installed on a network and detection is based on the traffic captured on the network. Therefore, this eliminated the need to execute intrusion detection on resource-constrained IoT devices.

IDS detection methods can be broadly classified into two types: signature-based IDS and anomaly-based IDS. Signature-based IDSs predefine the communication patterns of known attacks as attack signatures and execute intrusion detection by examining the similarity of captured traffic to the predefined attack signatures. However, signature-based IDSs cannot detect unknown attacks until IDS vendors release new attack signatures, namely, it is impossible to handle zero-day attacks since no signature represents the zero-day attacks. Anomaly-based IDSs, on the other hand, predefine the normal state of a monitored network and compare the traffic to this predefined normal state at the time of detection to determine whether there is a deviation from the normal state, usually caused by attacks. Therefore, an anomaly-based IDS can detect unknown attacks, whereas a signature-based IDS requires a signature update for the detection. However, there is a concern that anomaly-based IDSs may increase the false positive rate because normal observations may exceed the predefined normal range [4]. Considering that IoT networks generally consist of heterogeneous devices with different hardware and operating systems that lead to a diverse attacks compared with non-IoT networks [5], the use of anomaly-based IDS that can detect unknown attacks without updating signatures in IoT networks is more suitable.

A unique characteristic exists in that the amount of traffic data per network is limited even though a large number of devices generally exist in IoT networks. This results in a limited sample size for training intrusion detection models for IDS systems. Therefore, there are methods that utilize distributed learning for building and improving intrusion detection models in IDS. Combining distributed learning allows an anomaly-based IDS to collect attack-related samples from numerous networks, which enables the learning of intrusion detection models that can detect a variety of attacks, even with a small number of samples per network. However, distributed learning involves the direct exchange of learning data between the participating devices and the aggregation server, which raises privacy concerns. Another concern is that the direct exchange of learning data may consume significant communication resources in IoT networks and aggregation servers, which can potentially result in a considerable communication overhead.

Federated Learning (FL) [6] is an algorithm that builds a global model by aggregating model update information from clients while keeping the learning data distributed on the client side to address the aforementioned privacy and overhead concerns. In IoT networks, the combination of anomaly-based IDS and FL has a high affinity in terms of resource limitations, device quantity, device diversity, zero-day attack countermeasures, and privacy protection, and some integrated IDSs have been proposed [7,8]. These proposals leverage the characteristics of IoT networks and FL to address zero-day attacks. However, there remain challenges, e.g., it is unable to share attack information obtained from different device types, and there is no discussion on how to extract and label zero-day attacks.

In this paper, we introduce IDAC, a novel method that aggregates attack candidates extracted based on communication traffic from each IoT network using FL to address the issue of sharing attack information obtained from different device types. Attack candidate extraction is executed by applying outlier detection, which is an unsupervised learning method, to the entire network traffic. Subsequently, by training the extracted candidate attacks using novelty detection, the proposed method builds an intrusion detection model to classify whether another new input candidate is included in the learned candidate attacks. This approach allows it to realize an autonomous detection of zero-day attacks in environments with multiple device types.

The main contributions of this paper are as follows.

- Proposal of IDAC, an intrusion detection method that can be applied to zero-day attacks by sharing attack candidate information through FL to address the issues of the conventional methods that cannot label attack candidates autonomously.
- Confirmation that the proposed IDS with IDAC can achieve comparable detection performance against various attacks including zero-day attacks by suppressing false positives and missed detection in the extraction of attack candidates through a computer simulation-based evaluations using the BoTIoT dataset [9].
- Verification that sharing attack candidates can improve both attack detection performance and attack detection time.
- Confirmation that IDAC has the capability for real-time processing of incoming traffic by resolving the issue in the flow conversion process.

## 2. Related Work

This section describes related research on Intrusion Detection Systems (IDS) for zero-day attack detection and IDS using Federated Learning (FL).

### 2.1. IDS for Zero-Day Attack Detection

To address the issue that anomaly-based IDSs have a high FP rate, an intrusion detection method using an autoencoder is proposed to detect zero-day attacks with a high TP rate and a low FP rate [10]. In this method, the autoencoder is first trained by normal traffic data to build an intrusion detection model for IDS. Then, the learned intrusion detection model is applied to traffic data containing zero-day attacks to detect intrusion by novelty detection, which is a kind of anomaly detection algorithm. Novelty detection pre-learns the state of one class and examines whether new input data belongs to the learned class. Detection performance was evaluated using the CICIDS2017 and NSL-KDD datasets, and the method achieved high accuracy in detecting zero-day attacks. Furthermore, the method achieves a higher detection performance than the one using novelty detection executed by Online OC-SVM. However, the detection performance of the method against zero-day attacks relies on pre-learning the normal state, and the heterogeneity of devices in IoT networks prevents it from defining a normal state for each device in a real-world environment.

To address the above issue, an anomaly-based IDS that does not require defining normal states has been proposed for zero-day attacks. This method combines Subspace Clustering (SSC) and OC-SVM to execute highly accurate anomaly detection without prior knowledge [11]. SSC is an unsupervised clustering technique that uses SSC to form multiple subspace clusters using the feature spaces composed of network traffic converted into features. Clustering with SSC aggregates data points with similar features into a single subspace. Then, outlier detection is applied to each subspace using OC-SVM to extract zero-day attacks. Outlier detection is an anomaly detection algorithm that extracts outliers of input data. This enables highly accurate detection of complex zero-day attacks without defining normal states. The NSL-KDD dataset was used to evaluate the detection performance and the evaluation demonstrates higher performance than existing clustering methods.

A reinforcement learning-based method to improve detection performance under environments that are difficult to aggregate sufficient amount of traffic samples by generating traffic samples inside networks was also proposed in [12]. This method generates traffic samples based on captured traffic and improves the performance of the intrusion detection model for attacks including zero-day attacks based on samples manually labeled by professionals. The performance evaluations show that the F1 scores of zero-day attacks that are not included in the learning data exceed 0.7.

However, in IoT networks there is a concern that it may take time to generate clusters capable of extracting zero-day attacks with high accuracy since there are generally insufficient amounts of data to form clusters. Moreover, traffic sample generation cannot always generate appropriate data for learning since the generation is based on the past records. Namely, the number of data points in a single subspace may be too small, and this could be a cause of FP increase when applying OC-SVM. In addition, there are diverse attacks in IoT networks and manual labeling is not appropriate to cope with them due to its low adaptability. Therefore, FL-based distributed learning methods have been proposed to solve the issues.

### 2.2. IDS with Federated Learning

Anomaly-based IDSs face a high FP rate, and that becomes even more severe in IoT networks due to their traffic diversity [7]. This also means that it is difficult to build an anomaly-based detection model that can be applied to all behaviors in IoT networks with heterogeneous devices due to the high FP rate. To address the issue, intrusion detection models for individual device types using Gated Recurrent Units (GRUs) have been proposed. In addition, the model combines FL to efficiently utilize the small amount of data collected from each IoT network. The detection performance evaluation revealed that the method could achieve high detection performance under an environment including Mirai-infected IoT devices. The detection performance evaluation used traffic collected in an experimental environment with Mirai-infected IoT devices and demonstrated that the method has high detection performance. However, the method requires multiple devices for a single device type to improve detection performance, and the attack information cannot be shared among different device types. This limitation in attack information sharing may be an obstacle to anomaly detection since the case that different types of devices use common software exists, that is, individual detection model building is required even though there is a similarity in attack information.

The fact that the estimated total amount of data generated by IoT networks in the entire world is about 79.4 ZB makes it difficult to collect training data and build an intrusion detection model on a central server after installing IDSs on each IoT network due to network resource constraints [8]. Therefore, the paper pointed out that it is difficult to build an intrusion detection model by installing IDSs on each IoT network as well as collecting learning data centrally on a central server due to network resource constraints. Therefore, a method that learns zero-day attacks detected by each IoT edge device using a Deep Neural Network (DNN) and shares the learned models among devices using FL has been proposed. This method solves the aforementioned issue by gathering locally built intrusion model information at the central server and distributing updated model information to edge devices. N-BaIoT and Bot-IoT datasets were used to evaluate the detection performance. The detection performance evaluation demonstrated that an attack unknown to one device could be detected by other devices that learn similar attacks by sharing various types of attacks through FL. However, there is no discussion about internal traffic data labeling, which is required to label traffic data with zero-day attacks as attacks inside devices.

FL can efficiently share zero-day attack information and enable attack detection in each network. However, autonomous detection and sharing of attack information among devices in a shared environment with FL is impossible as explained. Therefore, this paper proposes a method to build an intrusion detection model by aggregating attack candidates using Online OC-SVM and shares the intrusion detection model by FL to realize autonomous detection of zero-day attacks in IoT networks regardless of device type.

## 3. IDAC: Intrusion Detection Based on Attack Candidate

### 3.1. System Overview

**Figure 1:**
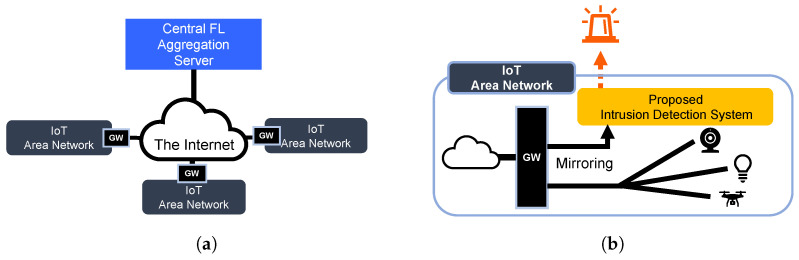
Assumed network environment. (**a**) Example of network configuration; (**b**) Installation example of IDS with IDAC.

To address the aforementioned issues that conventional intrusion detection methods fail to label captured traffic data autonomously and to share attack information among various kinds of devices, this paper proposes Intrusion Detection based on Attack Candidate (IDAC). IDAC is a novel approach for building intrusion detection models by aggregating attack candidates accepting a certain level of false positives (FP) with One-Class Support Vector Machine (Online OC-SVM) and sharing the intrusion detection models through Federated Learning (FL). This paper assumes an intrusion detection system based on IDAC is installed on each IoT network as shown in Figure 1a, and a central server is connected to facilitate FL among the networks. Network traffic is mirrored at the IoT Gateway (IoT GW), and IDS with IDAC receives the network traffic for intrusion detection within each IoT network as shown in Figure 1b.

**Figure 2:**
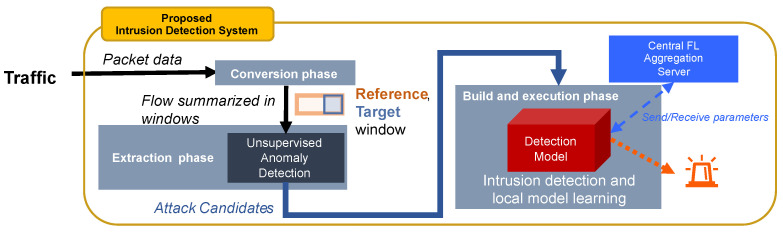
Intrusion detection process in IDS with IDAC.

Figure 2 depicts the intrusion detection process of IDAC installed on each network. IDS with IDAC executes intrusion detection sequentially each time the latest network traffic is inputted. The process from the input of network traffic to the detection of intrusions is carried out through the following phases:

1. Conversion phase: Convert traffic to flow;
2. Extraction phase: Extract attack candidates from flow;
3. Build and execution phase: Build detection model and execute intrusion detection;
4. Improvement phase: Improve detection models using FL.

The following subsections describe detailed procedures in each phase.

### 3.2. Conversion Phase

In this phase, time windows are created at a fixed interval for mirrored continuous real-time traffic. The set of packets existing within each time window is then converted into flow information, which is subsequently transformed into feature vectors. Based on the feature vectors of the flows, IDS with IDAC executes intrusion detection for each flow.

**Figure 3:**
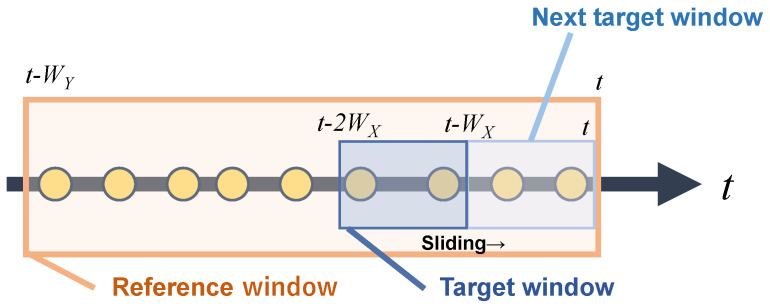
Creation of target window and reference window.

Since network traffic is continuously inputted into a conversion mechanism in real-time, detection target time windows of length $w_{X}$, denoted as $X_{t}$, are sequentially created as shown in Figure 3. This mechanism adopts a similar concept of a sliding window. The mechanism invokes flow conversion within a time window once the window is fully occupied and generates a new time window afterward. However, flow conversion concluded within $X_{t}$ may fail to capture the unique characteristics of long-term attacks that have more duration than $X_{t}$. Therefore, the mechanism simultaneously generates a reference time window $Y_{t}$ of length $w_{Y}\;(w_{Y}\geq w_{X})$ to refer to long-term characteristics. $Y_{t}$ is continuously updated to maintain a time series $y_{t-w_{X}+1},\cdots ,y_{t-1},y_{t}$, and is referenced at the point of flow conversion in $X_{t}$. Although it can achieve high detection accuracy with $w_{X}=w_{Y}$, processing real-time traffic under this condition leads to substantial resource consumption for intrusion detection. Reducing $w_{X}$ and $w_{Y}$ can minimize resource consumption but raises concerns about the inability to capture long-term characteristics. Therefore, setting $w_{Y}>w_{X}$ is essential to strike a balance between detection effectiveness and resource efficiency.

IDAC utilizes Argus [13], a tool designed for auditing network activity, to convert network traffic to flow information. Argus can transform traffic from network interfaces or files in the Packet Capture (pcap) format into network flow information. In the flow conversion process using Argus, packet data contained within the reference time window is transmitted to Argus, and Argus retrieves flow information for the packet data within the detection target time window. Subsequently, ten critical features that can be used for intrusion detection are extracted from the flow information. The selected features are listed in Table 1. The features to be extracted are based on those used in the creation of the BoTIoT [9] dataset as it identifies ten features that enable the most accurate detection through statistical methods.

After extracting the features, min–max normalization is applied to them. This process aims to enhance the classification performance in the Support Vector Machine (SVM) used for candidate extraction and intrusion detection by scaling the feature values to a fixed range. Given the need to process traffic in real-time in IDS with IDAC, normalization is executed by setting the maximum and minimum values for each feature based on the ordinal value ranges that they generally take. In this study, the definitions of the values for normalization are provided in Table 2.

### 3.3. Extraction Phase

In the extraction phase, unsupervised anomaly detection is executed on time series flow data converted in the previous phase. Anomaly detection is conducted by applying Outlier Detection within the latest preprocessed time series data $X_{t}$, thereby extracting outliers that are considered anomalies. One-class SVM is utilized for anomaly detection with parameters in Table 3. IDS with IDAC regards these outliers as autonomously extracted anomalous information and treats them as attack candidates.

However, to prevent attack flows from being continuously identified as attack candidates when the number of attack flows exceeds the number of normal flows in the targeted time series data, flows identified as outliers are recorded for a certain period and excluded from the detection target time series data. This approach helps in suppressing false positives. The exclusion applies to flows that the tuples (Protocol, Source Address, Destination Address, Source Port, Destination Port) are completely matched.

### 3.4. Build and Execution Phase

**Figure 4:**
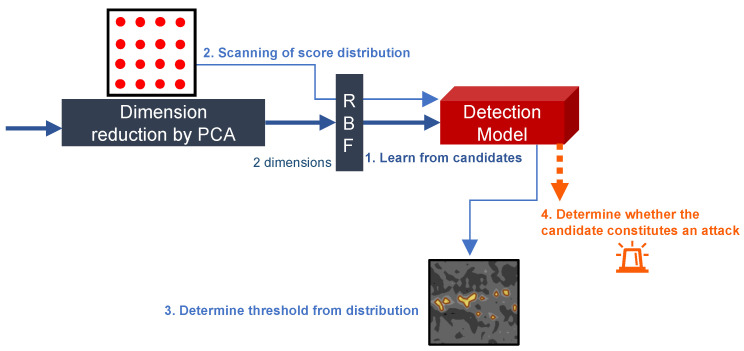
The conversion process of attack candidate prior to model building.

The attack candidate data require further transformation to build an intrusion detection model by aggregating the attack candidates extracted in the previous process. The outline of the data transformation process in this stage is shown in Figure 4. Intrusion detection is executed by determining whether new traffic is included in the learned attack candidates. Moreover, developing a model that supports attack information sharing among networks and online learning via FL is crucial. For the requirements to achieve the aforementioned objectives, algorithms that satisfy the following conditions are required.

 **Requirement 1:** A one-class classification algorithm that can determine whether the inference data represent an attack based on the learned attack candidates.
 **Requirement 2:** A parametric model whose form is predetermined and can be explained by its parameters.
 **Requirement 3:** Support online learning that allows for updates based solely on new data rather than batch learning to facilitate real-time traffic processing.
 **Requirement 4:** Capable of prioritizing intrusion detection processing on the most recent learning data to adapt to changes in attack characteristics.

In IDAC, a linear Online OC-SVM based on Stochastic Gradient Descent (SGD), which fulfills the aforementioned requirements, is employed to execute learning using attack candidates. Online OC-SVM, a machine learning technique derived from OC-SVM, is a parametric model, and thereby meets conditions Requirements 1 and 2. Moreover, its capability for online learning satisfies Requirement 3, and it also meets Requirement 4 by virtue of its online learning nature. Furthermore, being an online learning model allows faster learning and inference processing compared to traditional OC-SVM.

In Online OC-SVM, γ and ν are the primary hyperparameters, and the classification performance of models significantly varies based on the parameters. This approach ensures that the model is not only adaptable and efficient in real-time environments but also capable of continuous improvement and adjustment to emerging threat patterns.

However, since linear models cannot classify linearly inseparable data, it is challenging to build a high-accuracy classifier using Online OC-SVM alone after learning from attack candidates. Therefore, kernel approximation techniques are employed to map data that are linearly inseparable into a higher-dimensional feature space where linear classification is executed. Various approximation methods such as Nyström approximation and Random Fourier Features (RFF) have been proposed with their unique advantages. In this paper, IDAC uses RFF because it has compatibility with FL and does not require initial parameter sharing.

The threshold for anomaly scores varies depending on the learned attack candidates. Therefore, identifying the anomaly score distribution and determining a threshold that marks outliers in this distribution are essential steps after generating the model. However, comprehending the distribution in a feature space of three dimensions or more is challenging due to resource consumption. Thus, Principal Component Analysis (PCA) is used to reduce the information of each flow to a lower-dimensional space. IDAC reduces the feature space to two dimensions using PCA after extracting features listed in Table 1. The parameters for PCA are predetermined as IDAC executes real-time traffic processing. Although the dimensionality is arbitrary, the dimensionality of the reduced feature space is set to two to facilitate swift scanning of the anomaly score distribution.

The search for anomaly scores is conducted after the dimensionality reduction and kernel approximation processes, followed by the intrusion detection model building using Online OC-SVM. The search for anomaly scores initially sets a grid of 50×50 points within the possible value range on the two-dimensional feature space X×Y. Then, a set of anomaly scores S represented by Equation (1) is created by inputting the score $S(x_{i},y_{i})$ at $\left(x_{i},y_{i}\right)$ into the built intrusion detection model.

(1) $$S=\{S\left(x_{i},y_{i}\right)\;|\;\left(x_{i},y_{i}\right)\in X\times Y\}.$$

Subsequently, the *z*-score $z_{i}$ for each anomaly score is computed according to Equation (2) assuming that S follows a normal distribution.

(2) $$z_{i}=\frac{S(x_{i},y_{i})-\mu }{\sigma },$$

where μ is the mean of S and σ is the standard deviation of S. Finally, θ is determined according to Equation (3).

(3) $$\theta =\min\{S\left(x_{i},y_{i}\right)\;|\;z_{i}>2,\;S\left(x_{i},y_{i}\right)\in S\}.$$

After conducting the intrusion detection based on the threshold θ, the local intrusion detection model is refined through online learning with the identified attack candidates. This update process is referred to as local aggregation. This step is required to update θ to reflect the altered anomaly scores distribution of the model after the model update. In the context of enhancing the intrusion detection model with FL, a phase of retraining the local model with the gathered attack candidates is required. This retraining occurs after the last enhancement and before the commencement of the next enhancement, and thus preserving the attack candidates collected during this interval becomes essential for the next model updates.

### 3.5. Improvement Phase

In IDS with IDAC, the central server receives the model parameters of local intrusion detection models from each local node to aggregate them and then redistributes the aggregated model parameters back to each local not for model update every time a certain local model update occurs. This update process is referred to as global aggregation. Upon receiving the aggregated new model parameters, clients overwrite their model parameters with the received ones to synchronize with the parameters of the global model and then resume online learning. The aggregation is based on the FedAvg algorithm that incorporates a momentum strategy into FedAvgM [14].

The global aggregation is executed in a synchronous manner that temporarily pauses the local aggregation with a certain number of attack candidates within each network participating in the intrusion detection model improvement and resumes the intrusion detection process after the global aggregation using FL is completed. Initially, the aforementioned detection and learning processes are repeated on each network after initializing the local intrusion detection models in the central server and each network. Subsequently, a request for aggregation is sent to the central server once learning with a certain number of attack candidates has been conducted. Upon receiving the request, the central server collects the current parameters of the local models from each network to aggregate them and conducts a process that receives the aggregated parameters and applies them to the local models for *n* rounds.

## 4. Performance Evaluation

### 4.1. Simulation Setups

In this subsection, detection and processing performance evaluations based on existing datasets are conducted to confirm the effectiveness and feasibility of IDAC. Both local and global aggregation of attack candidates by IDAC is confirmed to work as designed for the feasibility evaluation. Moreover, we implement IDAC on low-power single-board computers that are commonly used in practical IoT networks and measure the real-time traffic processing performance.

We used the BoTIoT dataset [9] to evaluate the detection and processing performance. IDS with IDAC is implemented using Python (version 3.10.12) using the Scikit-learn [15], and Flower [16] libraries. Table 4 shows the required parameters for IDAC.

#### 4.1.1. Detection Performance Evaluation

In the detection performance evaluation, we set up the following two scenarios to assess detection performance:

1. Scenario 1
  Scenario 1 assumes that a single IoT device exists in a network and measures detection performance after the device executes local aggregation for each attack type. The evaluation is conducted using grid search by varying $\gamma_{2}$, $\nu_{2}$, $W_{X}$, and $W_{Y}$ based on the combinations shown in Table 5. The data ranges of BoTIoT used for this evaluation are shown in Table 6.
2. Scenario 2
  Scenario 2 assumes that multiple IoT networks exist in the environment, and we measure the detection performance when each IoT network detects the same type of attack and executes global aggregation. This scenario also evaluates the performance of the proposed method with FL and without FL (non-FL) as in Scenario 1. In the evaluation of non-FL, Network1 is used for the dataset whereas the proposed method with FL uses both Network1 and Network2. Scenario 2 also varies the parameters as in Scenario 1, and the date ranges are shown in Table 6 and Table 7.

The calculation of evaluation metrics is based on the confusion matrix obtained from the classification results of intrusion detection. The components of the confusion matrix are defined as follows:

 **True Negative (TN)** The number of flows that are not classified as a single attack when the flows are not attacks.
 **False Positive (FP)** The number of flows that are classified as attacks at least once by the intrusion detection process when the flows are attacks.
 **False Negative (FN)** The number of flows that are not classified as a single attack when the flows are attacks.
 **True Positive (TP)** The number of flows that are classified as attacks at least once by the intrusion detection process when the flows are attacks.

In this evaluation, we use the following three items as evaluation metrics.

 **True Positive Rate (TPR, Recall)** The Recall *R* represents the proportion of flows that are actually attacks and are correctly distinguished as such by the intrusion detection results. Here, $F_{N}$ denotes the number of flows classified as FN and the equation to calculate $F_{N}$ is shown in Equation (4).
  (4) $$R=\frac{T_{P}}{T_{P}+F_{N}}$$
 **True Negative Rate (TNR, Specificity)** The Specificity *S* represents the proportion of flows that are actually normal and are correctly distinguished as such by the intrusion detection results. Here, $T_{N}$ denotes the number of flows classified as TN and the equation to calculate $T_{N}$ is shown in Equation (5).
  (5) $$S=\frac{T_{N}}{T_{N}+F_{P}}$$
 **F1 Score:** The F1 Score is is the harmonic mean of Precision *P* and Recall *R*, which ranges 0 to 1.0. The equation to calculate F1 is shown in Equation (6).
  (6) $$F1=\frac{2PR}{P+R}$$

The equation to calculate *P* is shown in Equation (7), where $T_{P}$ denotes the number of flows classified as TP and $F_{P}$ denotes the number of flows classified as FP.

(7) $$P=\frac{T_{P}}{T_{P}+F_{P}}$$

Additionally, the time taken from attack flow occurrence until it is detected is measured to show the swiftness of detection.

Among the parameters that need to be set, the following parameters are set beforehand based on preliminary experiments, and other parameters are individually set in the experiments for each scenario. The dimension of RFF is set to 200, the parameters for OC-SVM are set to $\left(\theta_{1},\gamma_{1},\nu_{1}\right)=\left(-0.0005,10,0.2\right)$, and the random state is set to 42.

#### 4.1.2. Processing Performance Evaluation

The processing performance evaluation was conducted on the experimental testbed whose specification is shown in Table 8 with the dataset listed in Table 9. The parameters were set to $\left(W_{X},W_{Y},\gamma_{2},\nu_{2}\right)=\left(10,120,5,0.5\right)$, and the other parameters are the same as those used in the detection performance evaluation experiments.

In this evaluation, we measured the time required by IDAC for flow conversion, attack candidate extraction, model improvement, and attack detection 10 times. The evaluation metric was the time required for processing in seconds.

### 4.2. Evaluation Results

#### 4.2.1. Detection Performance Evaluation

Table 10 shows the combination of parameters that resulted in the highest average values of the TPR and TNR for each attack type in Scenario 1. Table 11 shows the parameters and detection performance for each attack type in Scenario 2. The parameters in the table are those with the highest average TPR and TNR values in Network1 for each attack type. Here, the F1 score does not indicate classification performance for normal flows, and thus we show both TPR and TNR scores.

Table 12 shows intrusion detection time for flows that were actually the attacks and were identified as such. Table 12 shows the time required for the detection with the parameters that can achieve the highest detection performance in Scenario 1, where STD represents the standard deviation. Note that the actual process of non-FL has small differences in Scenario 1 and Scenario 2 and thus this paper uses the result of Scenario 1 in this evaluation.

#### 4.2.2. Processing Performance

**Figure 5:**
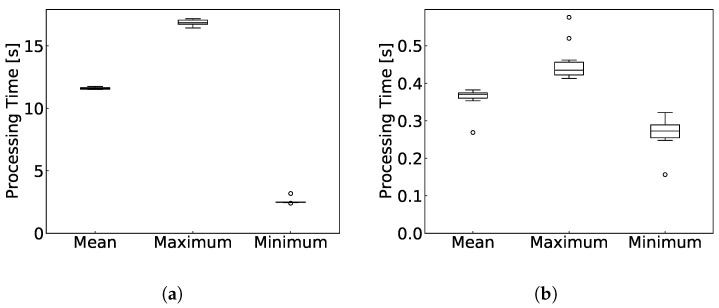
Results of processing performance evaluation. (**a**) Time required to convert packet data into flow information; (**b**) Time required for intrusion detection based on flow information.

Figure 5 illustrates the average, maximum, and minimum time duration required for the processing. Figure 5a shows the time required to convert packet data into flow information using Argus, and Figure 5b shows the time required to perform intrusion detection based on flow information using the proposed method.

**Figure 6:**
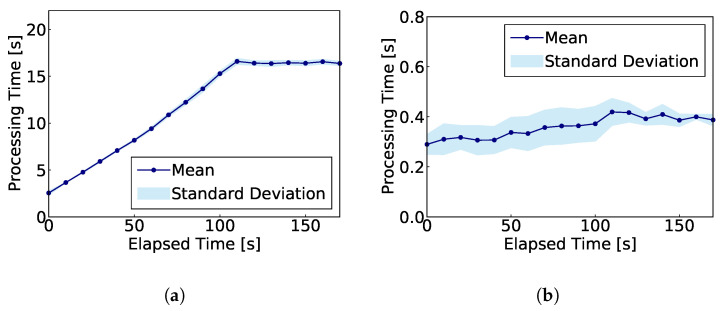
Transition of processing time in processing performance evaluation. (**a**) Time required to convert packet data into flow information; (**b**) Time required for intrusion detection based on flow information.

Figure 6 shows the progression of processing times in the processing performance evaluation. Figure 6a shows the progression of time taken to convert packet data into flow information, and Figure 6b shows the progression of time taken to perform intrusion detection based on flow information using IDAC.

### 4.3. Discussion

The results in Table 10 for Scenario 1 show that the F1 score for all attack types consistently exceeded 81%. Additionally, the incidence of false positives from misclassified normal flows is under 9% since the TNR surpasses 0.91. This evidence confirms that aggregating autonomously extracted attack candidates enables IDAC to achieve intrusion detection accuracy on par with traditional approaches, and effectively minimize false positives and misses in the attack candidate extraction phase. Moreover, IDAC can detect zero-day attacks since the attack candidates are outliers that are autonomously extracted from recent traffic. However, parameters for the best detection performance vary according to attack types, especially for $W_{X},W_{Y}$. This is because the values demonstrating high detection performance significantly differ among attack types, and this suggests the necessity of final detection decisions based on the results from detectors set with multiple $W_{X},W_{Y}$ values.

Table 11 shows that the proposed method improves the TPR, TNR, and F1 score for OSScan, Keylogging, DDoS, and data exfiltration attacks in FL-Network1 in comparison to non-FL by including Network2 information in the learning process. This implies that aggregating similar attack candidates from networks using FL can improve detection performance. On the other hand, the participation of Network2 results in a decrease in TPR and F1 scores for ServiceScan attacks. In addition, Table 11 also shows that the F1 score for data exfiltration and TNR for DDoS in Network2 are low compared to the others. This issue is caused by the performance degradation of the learning model trained with different data distributions in FL.

Table 12 clearly shows a reduction in the time required to detect DDoS, OSScan, ServiceScan, and keylogging attacks. Table 12 shows that the proposed method shortens the required time for detecting OSScan, Keylogging, DDoS, and data exfiltration in FL-Network1 by accompanying networks in the learning process with the parameters explained in the Table 12 This reduction indicates that sharing attack candidates allows quicker attack detection. Thus, the proposed method can aggregate more attack candidates in a shorter time compared with the non-FL method. In other words, a more accurate intrusion detection model can be established in a short time and the proposed method enables swift attack detection by sharing the model among networks. However, the time required to detect data exfiltration attacks has increased. As previously mentioned, the detection accuracy for data exfiltration attacks significantly decreased due to the divergence of the intrusion detection model, and this also affects the time required for detection.

Figure 5 reveals that the time required for intrusion detection processing for a single detection target time window exceeded $W_{X}$. Thus, executing real-time traffic processing in the environment detailed in Table 8 presents significant challenges. However, Figure 6a,b demonstrate that a considerable portion of the intrusion detection processing time is allocated to the process of converting packet data into flow information in Argus. In other words, the intrusion detection processing time itself is shorter than $W_{X}$, and improving the conversion process makes real-time traffic processing highly feasible. This improvement in the conversion process can be achieved by introducing another conversion method that has a higher processing performance to handle real-time flows.

An IDS with IDAC in a practical IoT network environment enables the extraction of attack candidates on any IoT network regardless of network characteristics even though the aforementioned countermeasures against performance degradation and the flow information conversion process need to be addressed. Given the current existence of many operational IoT networks and the fact that IDAC performs detection based on potential attack candidates, more attack features can be collected and aggregated in a shorter period of time as the number of network participants increases. Consequently, IDAC enables autonomous attack detection with low FP and FN rates for attacks that exploit vulnerabilities discovered daily with each IoT network collaboration. This significantly contributes to preventing the spread of damage caused by cyberattacks within the IoT ecosystem.

The current limitation of IDAC is that the detection performance changes depending on network features and attack types. Therefore, the detection performance may face performance degradation when the parameters for detection are not appropriate for the environment. Changing the parameters according to the network features and attack types helps to maintain a certain level of detection performance. However, this would not guarantee to maintain the performance and the detection performance degradation is inevitable in some cases. Moreover, IDAC, which is a single-detector-based method, may face difficulties in choosing appropriate parameters across all participating networks. One of the solutions to this limitation is to use a multiple-detector-based method to set appropriate parameters individually. For this, more evaluations and investigations for IDAC are required to analyze the behavior of IDAC.

## 5. Conclusions

In this paper, we proposed a novel method for intrusion detection based on attack candidates to solve the existing challenges in detecting zero-day attacks using FL in IoT networks. IDS with IDAC deemed suspicious traffic extracted by an unsupervised anomaly detection algorithm in each IoT network as attack candidates and aggregates these candidates both within and among networks. This aggregation of attack candidates detected within and among IoT networks enables autonomous intrusion detection based on the aggregated candidates. The performance evaluations have demonstrated that IDAC achieves comparable detection performance to conventional methods, enabling real-time intrusion detection even on devices with limited computing resources. Therefore, we conclude that IDAC realized autonomous intrusion detection based solely on the features of similar attacks simultaneously observed on other IoT networks using FL. Although the IDAC may degrade its detection performance on specific attack types as previously discussed, using multiple detectors and employing suitable hyperparameters for each detector can overcome the issue.

## Figures and Tables

**Table 1:** Features extracted from the flow in Argus.

Feature	Description
srate	Source packets per second
drate	Destination packets per second
rate	Packets per second
max	Maximum duration of aggregated records
state	Transaction state
mean	Average duration of aggregated records
min	Minimum duration of aggregated records
stddev	Standard deviation of aggregated duration times
flgs	Flow state flags seen in transaction
seq	Sequence number

**Table 2:** Definition of generally assumable values used for normalization.

Feature	Minimum Value	Maximum Value
srate	0	7500
drate	0	7500
rate	0	100,000
max	0	5
state	0	1
mean	0	5
min	0	5
stddev	0	2
seq	0	800

**Table 3:** Parameters for OC-SVM used in anomaly detection.

	Parameter	Value
Common	Random State	42
OC-SVM	γ	$1.0\times 10^{-4}$
	ν	0.01
	Threshold	–$3.0\times 10^{-4}$

**Table 4:** Parameters required for IDAC.

	Parameter	Description
Common	Random State	Seed value
RFF	Dimension	Dimensionality in RFF
Window	$W_{X}$	Length of the target window
	$W_{Y}$	Length of reference window
OC-SVM	$\gamma_{1}$	RBF kernel parameter
	$\nu_{1}$	Fraction of training error
	$\theta_{1}$	Threshold for anomaly scores
Online OC-SVM	$\gamma_{2}$	RBF kernel parameter via RFF
	$\nu_{2}$	Fraction of training error
	$\theta_{2}$	Threshold for anomaly score
Federated Learning	Round	Number of rounds
	Momentum	Parameter of FedAvgM
	Learning Rate	

**Table 5:** Parameters to be modified in Scenario 1 and Scenario 2.

$\gamma_{2}$	$\nu_{2}$	$W_{X}$	$W_{Y}$
0.1	0.001	1	30
1	0.01	5	60
5	0.1	10	120
10	0.2		
20	0.5		
25			

**Table 6:** Overview of the dataset used for evaluation in Scenario 1 and Scenario 2 (Network1).

Attack Type	Time Range	Number of Normal Flows	Number of Attack Flows
DDoS	04-06-2018 09:01:04–09:04:55	8	4136
Data exfiltration	18-06-2018 01:09:22–01:11:01	10	113
OSScan	05-21-2018 04:05:05–04:21:16	213,147	651
ServiceScan	05-15-2018 00:27:11–00:58:15	310,286	1377
Keylogging	06-19-2018 03:59:03–04:07:18	121	687

**Table 7:** Overview of the dataset used for evaluation in Scenario 2 (Network2).

Attack Type	Time Range	Number of Normal Flows	Number of Attack Flows
DDoS	04-06-2018 09:04:55–09:08:17	9	7013
Data exfiltration	06-18-2018 01:11:01–01:12:33	8	2
OSScan	05-21-2018 04:20:46–04:32:28	785,185	2671
ServiceScan	05-15-2018 00:58:15–01:31:32	71,610	1430
Keylogging	06-19-2018 04:07:18–04:15:24	52	713

**Table 8:** Experimental environment for processing performance evaluation.

Component	Specification
Board	Raspberry Pi 3 Model B
SoC	Broadcom BCM2837RIFBGT
CPU	ARM Cortex-A53 (Utilizing 1 out of 4 cores)
RAM	1 GB
OS	Raspberry Pi OS 11 (bullseye)

**Table 9:** Overview of the dataset used for processing performance evaluation.

Attack Type	Time Range	Total Flow Count	Total Window Count
Data exfiltration	2018-06-18 01:09:22–01:11:01	548	18

**Table 10:** Parameters and detection performance for each attack type in Scenario 1.

Attack Type	$\gamma_{2}$	$\nu_{2}$	$W_{X}$	$W_{Y}$	TPR	TNR	F1
DDoS	10	0.5	10	120	0.89	1.00	0.94
Data exfiltration	10	0.5	5	30	0.81	1.00	0.89
OSScan	10	0.5	10	30	0.92	0.91	0.96
ServiceScan	10	0.001	10	30	0.69	1.00	0.81
Keylogging	5	0.5	5	60	0.95	0.98	0.97

**Table 11:** Parameters and detection performance for each attack type in Scenario 2.

Attack Type	$\gamma_{2}$	$\nu_{2}$	$W_{X}$	$W_{Y}$	Non-FL			FL-Network1			FL-Network2		
					TPR	TNR	F1	TPR	TNR	F1	TPR	TNR	F1
DDoS	20	0.2	5	30	0.99	0.36	1.0	1.0	0.7	1.0	0.99	0.6	0.99
Data exfiltration	10	0.5	5	60	0.77	0.64	0.85	0.8	0.8	0.88	0.33	0.73	0.29
OSScan	5	0.5	10	120	0.93	0.89	0.96	0.94	1.0	0.97	0.93	0.87	0.96
ServiceScan	10	0.2	1	30	0.9	0.46	0.95	0.79	0.62	0.88	1.0	0.56	1.0
Keylogging	5	0.5	5	30	0.89	0.52	0.9	0.93	0.96	0.96	0.79	0.93	0.88

**Table 12:** Comparison of detection times in Scenario 1 and Scenario 2.

Attack Type	Non-FL [s]		FL-Network1 [s]		FL-Network2 [s]	
	Mean	STD	Mean	STD	Mean	STD
DDoS	7.14	18.16	4.96	11.01	8.30	12.33
Data exfiltration	7.49	36.27	32.53	22.53	25.03	25.03
OSScan	0.43	2.06	0.23	1.52	0.65	2.49
ServiceScan	1.76	4.62	0.01	0.28	3.26	21.56
Keylogging	2.37	7.56	2.21	6.98	0.83	12.13

## Data Availability

The dataset used for this article is available online at https://research.unsw.edu.au/projects/bot-iot-dataset, accessed on 25 February 2024.
